# The Wnt/β-catenin signaling pathway inhibits osteoporosis by regulating the expression of TERT: an in vivo and in vitro study

**DOI:** 10.18632/aging.205136

**Published:** 2023-10-19

**Authors:** Yuanqing Cai, Huijun Sun, Xingyu Song, Jianyu Zhao, Dong Xu, Mozhen Liu

**Affiliations:** 1 Department of Orthopaedics, The First Affiliated Hospital, Dalian Medical University, Xigang, Dalian 116011, China; 2 Department of Clinical Pharmacology, College of Pharmacy, Dalian Medical University, Lvshunkou, Dalian 116044, China

**Keywords:** osteoporosis, Wnt signaling pathway, TERT, H_2_O_2_, bilateral ovariectomy

## Abstract

Our study was performed to investigate whether the Wingless and int-1 (Wnt) signaling pathway promotes osteogenic differentiation and inhibits apoptosis in bone marrow mesenchymal stem cells (BMSCs) by regulating telomerase reverse transcriptase (TERT) expression. An in vivo model of osteoporosis (OP) in C57BL/6J mice by bilateral ovariectomy (OVX) and an in vitro model of H2O2-induced BMSCs were established separately. Western blotting was used to detect the expression of the pathway-related proteins TERT, β-catenin, and phosphorylated-glycogen synthase kinase-3beta (p-GSK3β)/GSK3β, the osteogenic-related markers osteopontin (OPN), bone morphogenetic protein 2 (BMP2), and runt-related transcription factor 2 (Runx2), and the apoptosis-related indicators B-cell lymphoma-2 (Bcl-2) and BAX. Osteoblastic phenotypes were also evaluated by alkaline phosphatase (ALP) staining and serum ALP activity assays. Osteogenic differentiation phenotypes in mice were verified by H&E staining, micro-CT, and parameter analysis of the femur. Western blotting results showed that the expression of the pathway-related proteins TERT, β-catenin, p-GSK3β/GSK3β was reduced in OVX mice and H2O2-induced BMSCs, accompanied by downregulated protein expression of osteogenic-related markers and antiapoptotic indicators and upregulated protein expression of apoptotic proteins compared to those in the control group. Mechanistic studies showed that the activation of Wnt signaling pathway in BMSCs promoted β-catenin translocation to the nucleus, as verified by immunofluorescence and facilitated colocalization between β-catenin and TERT, as verified by double-labeling immunofluorescence, thereby promoting osteogenic differentiation and reducing apoptosis. In summary, our experiments confirmed that the GSK3β/β-catenin/TERT pathway could regulate the osteogenic differentiation and apoptosis of BMSCs and that TERT might be a promising target for the future treatment of osteoporosis.

## INTRODUCTION

Osteoporosis (OP) is an orthopedic disease that is likely to occur in middle-aged and elderly people and imposes a great burden on individuals and society. Osteoblasts derived from bone marrow mesenchymal stem cells (BMSCs) drive bone formation, osteoclasts derived from hematopoietic stem cells drive bone resorption, and OP is the result of the disruption of the dynamic balance of these two cell functions [1, 2]. Patients with OP have lower bone density than healthy people, so these patients have a higher risk of fragility fractures and significantly higher mortality rates in severe cases. Studies in recent years have shown that osteoarthritis disease-related targets, such as HIF-1α, also play an important role in the development of osteoporosis [3, 4]. The existing drugs for the treatment of osteoporosis, including bisphosphonates, parathyroid hormone, and hormone replacement therapy, have some effects in delaying and treating osteoporosis [5, 6], but their routine long-term use as anti-osteoporosis drugs is partially limited by side effects such as organ damage, gastrointestinal discomfort, and safety issues [7]. Therefore, finding new therapeutic targets is necessary for the future treatment of osteoporosis.

BMSCs are common multipotent stem cells that can differentiate toward osteogenesis, chondrogenesis, and lipogenesis [8], and stem cell differentiation is regulated by multiple signaling pathways, including the canonical Wingless and int-1 (Wnt) pathway and the transforming growth factor beta (TGF-β)/bone morphogenetic protein (BMP) signaling pathway [9, 10]. The Wnt/β-catenin signaling pathway is indispensable for regulating human skeletal development and maintaining bone homeostasis *in vivo* [11–13]. In the canonical Wnt pathway, glycogen synthase kinase-3beta (GSK3β), Axin, and APC form a destruction complex that phosphorylates and degrades β-catenin. When the Wnt pathway is activated, GSK3β is degraded to p-GSK3β, which means that the destruction complex cannot be formed, and β-catenin is stabilized and translocated to the nucleus to promote osteogenic differentiation of BMSCs by binding to transcription factors such as T-cell factor 4 (TCF-4) and lymphoid enhancer-binding factor 1 (LEF-1) to upregulate the expression of target genes such as runt-related transcription factor 2 (Runx2) [14–18]. Lithium chloride (LiCl) has been commonly used for decades to treat bidirectional affective disorders [19]. As an inhibitor of GSK3β, LiCl activates the Wnt signaling pathway and promotes osteogenic differentiation of stem cells. Specifically, LiCl promotes the expression of Wnt pathway-related target genes by preventing the conversion of β-catenin to its phosphorylated form and facilitating its nuclear translocation [15, 20].

At the end of eukaryotic chromosomes, there is a cap-shaped structure, known as the telomere, which is continuously shortened during DNA replication, thus causing cellular senescence and apoptosis. Telomerase can counteract this phenomenon by continuously increasing the length of the telomere to compensate for the shortening of telomeres during DNA replication, thus increasing the cell survival cycle [21]. Telomerase reverse transcriptase (TERT) is an important catalytic subunit that is indispensable for telomerase to perform its telomere replication function, and the higher the expression of TERT is, the greater the ability of telomerase to function [22]. Decreased TERT activity and reduced osteogenic capacity in aging-related diseases such as osteoporosis caused by aging and sex hormone deficiency, as well as the positive effect of telomerase gene therapy on osteoporosis, have been reported [23, 24].

In two types of human cancer cells, β-catenin enables cancer development by occupying the promoter of TERT and thus upregulating TERT expression [25]. It has also been shown that TERT interacts with Brahma-related gene 1 (BRG1) to occupy specific chromatin sites of Wnt/β-catenin target genes, thereby regulating the expression of Wnt pathway target genes [26]. However, the function and regulatory network of TERT-dependent signaling in OP remain largely unknown. Therefore, this experiment was performed to investigate the phenotypic effects of TERT on the osteogenic differentiation and apoptosis of BMSCs and the specific mechanism underlying TERT regulation by the Wnt/β-catenin signaling pathway.

In the present experiment, both *in vivo* and *in vitro* results verified that the GSK3β/β-catenin/TERT pathway modulates osteogenic differentiation and apoptosis, thus exerting an anti-osteoporotic effect. The mechanistic study showed that when the Wnt pathway was activated, β-catenin nuclear translocation was increased, and the interaction between β-catenin and TERT was increased in the nucleus, which resulted in increased expression of TERT in BMSCs. Our experiments confirmed the promoting effects of TERT on osteogenic differentiation and the inhibitory effects on apoptosis of BMSCs both *in vitro* and *in vivo*, providing a new idea for the future treatment of osteoporosis.

## RESULTS

### LiCl reverses bone loss in OVX mice

**Figure 1:**
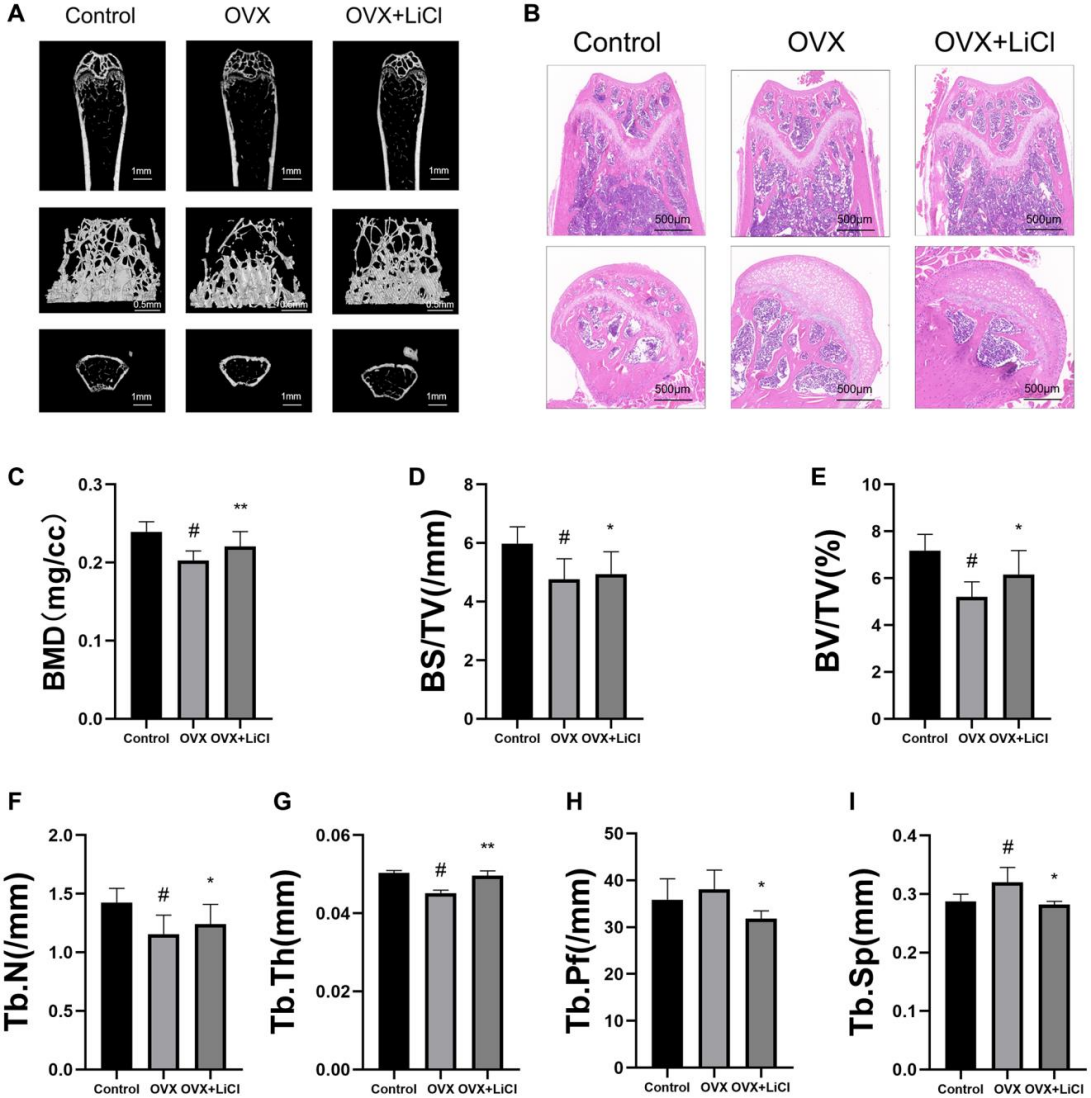
**LiCl reverses bone loss in OVX mice.** (**A**) Representative 3D micro-CT images of distal femurs from three groups (Sham surgery groups, OVX groups, OVX+LiCl-treated groups) were performed after eight-week surgery. (**B**) H&E staining was performed for evaluating the trabecular bone of distal femurs from each group. (**C**–**I**) Corresponding parameters showing the treatment of osteoporosis by LiCl therapy. Abbreviations: BV/TV: bone volume/tissue volume; Tb.N: trabecular number; Tb.Sp: trabecular spacing; BMD: bone mass density; Tb.Th: trabecular thickness; BS/BV: bone surface area/bone volume; Tb.PF: trabecular pattern factor. *n* = 6. All results are performed as mean ± SD. ^#^*P* < 0.05 vs. control group, ^*^P < 0.05, ^**^P < 0.01 vs. OVX group.

To investigate whether LiCl could improve bone loss in OVX mice, an osteoporosis model was established by ovariectomy in mice. The mice were sacrificed after 8 weeks of OVX, and their femurs were taken for micro-CT and hematoxylin and eosin (H&E) staining. Micro-CT showed that the trabeculae in the femurs of OVX mice were sparse and less continuous than those in the femur of control mice, while the application of LiCl restored the number and continuity of the trabeculae (Figure 1A). H&E staining similarly showed that the application of LiCl prevented bone loss caused by OVX in mice (Figure 1B). Parameter analysis of the femoral bone in mice showed a significant decrease in BV/TV, Tb.N, BMD, Tb.Th, and BS/TV and a significant increase in Tb.Pf and Tb.Sp in OVX mice compared to the control group, and the application of LiCl reversed these changes (Figure 1C–1I). The results indicated that the GSK3β inhibitor could reverse the reduction in osteogenic differentiation ability in mice caused by OVX.

### LiCl reverses the inactivation of Wnt pathway, increases osteogenic capacity, and reduces apoptosis levels in OVX mice

**Figure 2:**
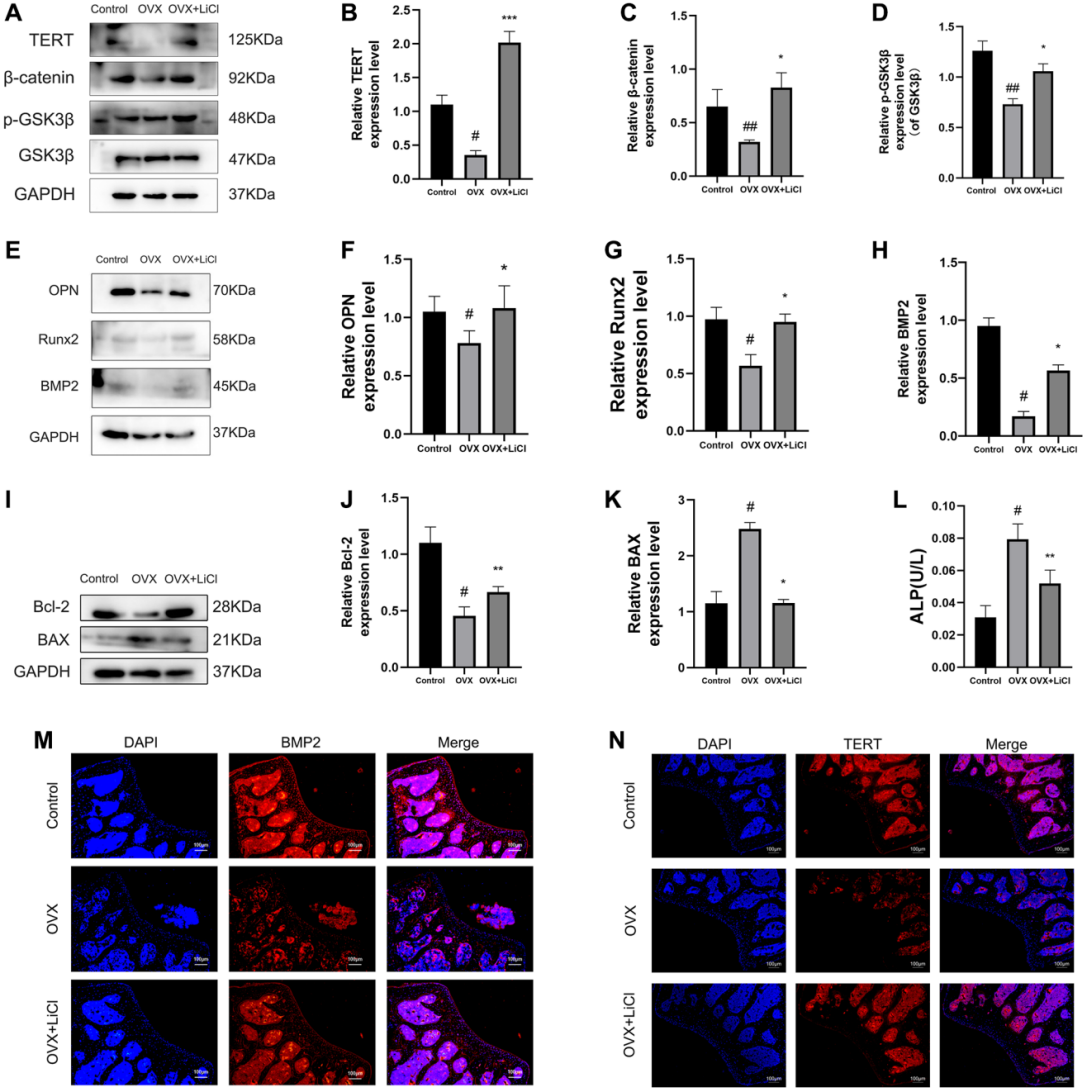
**LiCl reverses the inactivation of Wnt pathway, increases osteogenic capacity, and reduces apoptosis levels in OVX mice.** (**A**–**K**) Western blotting and quantification of TERT, β-catenin, p-GSK3β/GSK3β, OPN, Runx2, BMP2, Bcl-2, and BAX expression levels in the sham, OVX, and OVX+LiCl group mice. (**L**) ALP activity detected by ALP activity assay kit in three groups of mice. (**M**, **N**) The representative pictures of immunofluorescence staining (original magnification, ×400) and their analyses showed the expressions of BMP2 and TERT in mice femurs. All results are performed as mean ± SD. ^#^*P* < 0.05, ^##^*P* < 0.01 vs. control group, ^*^P < 0.05, ^**^P < 0.01, ^***^P < 0.005 vs. OVX group, *n* = 6.

The biological functions of TERT, β-catenin, and GSK3β in osteoporosis and the effects of LiCl on their expression have not been explored. The results showed that the protein expression of TERT and β-catenin and the p-GSK3β/GSK3β ratio were downregulated in the femoral tissues of the OVX mice compared to those in the control group, as measured by western blotting, while the trend of the above protein changes in mice in the LiCl gavage group was reversed (Figure 2A–2D). The expression of the osteogenic markers osteopontin (OPN), Runx2, and bone morphogenetic protein 2 (BMP2) was also decreased in the OVX group and increased in the LiCl-treated mice (Figure 2E–2H), indicating that the protein expression levels of TERT and β-catenin and the p-GSK3β/GSK3β ratio were positively related to the protein expression of osteogenic markers. The above results were also confirmed in human bone tissue samples, as detailed in Supplementary Figure 1. Then, the apoptosis level in bone tissue was determined. As shown in Figure 2I–2K, decreased expression of the anti-apoptotic protein B-cell lymphoma-2 (Bcl-2) and increased expression of the pro-apoptotic protein BAX, measured by western blotting in the femur tissue, were observed in OVX group mice, while the application of LiCl antagonized these results.

Meanwhile, the serum alkaline phosphatase (ALP) activity assay result, which indicates high bone turnover and osteoporosis in mice, showed that the serum ALP activity of mice in the OVX group was increased, while that of mice in the LiCl gavage group was decreased (Figure 2L) [27]. Consistent with the above results, immunofluorescence staining showed similar results for the expression of TERT and BMP2 (Figure 2M, 2N). The above results suggested that the GSK3β inhibitor could upregulate TERT protein expression, reverse the inactivation of the Wnt/β-catenin pathway, reduce osteogenic capacity, and increase apoptosis levels in OVX mice.

### Validation of the effects of TERT on osteogenic differentiation and apoptosis in BMSCs

**Figure 3:**
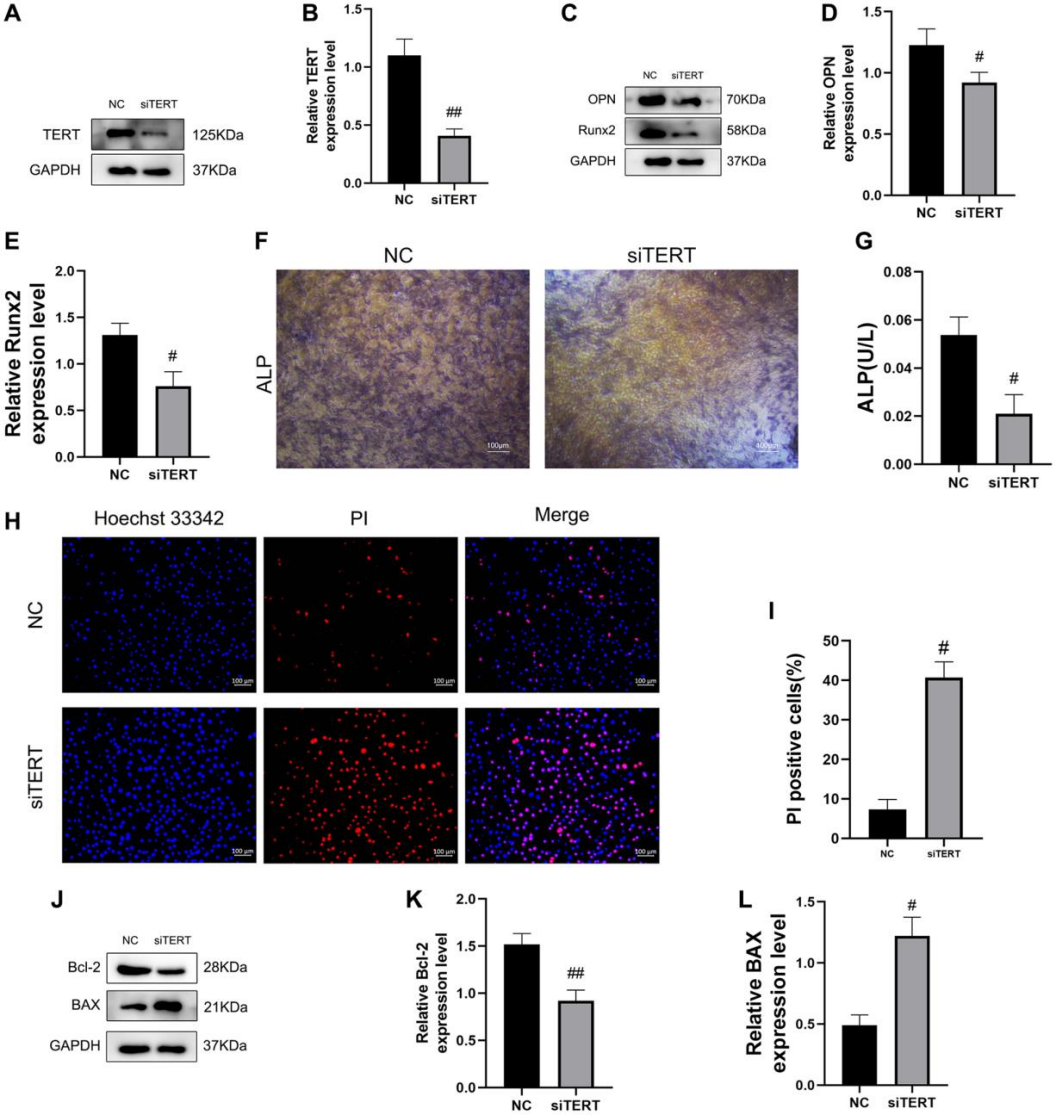
**Validation of the effect of TERT on osteogenic differentiation and apoptosis in BMSCs.** (**A**, **B**) The siRNA-mediated depletion of TERT assessed by western blotting. (**C**–**E**) The specific proteins related to osteogenesis differentiation detected by Western blotting, including OPN and Runx2. (**F**) ALP staining in NC group and TERT-depleted BMSCs. (**G**) ALP activity detected by ALP activity assay kit in NC group and TERT-depleted BMSCs. (**H**, **I**) The Hoechst 33342/PI staining and quantification results. (**J**–**L**) The specific proteins related to apoptosis detected by Western blotting, including Bcl-2 and BAX. All results are performed as mean ± SD. ^#^*P* < 0.05, ^##^*P* < 0.01 vs. NC group. *n* = 6.

To verify the effect of TERT on osteogenic differentiation and apoptosis in BMSCs, we transfected small interfering RNA of TERT into BMSCs to reduce its expression. Western blotting verified the successful knockdown of TERT in BMSCs (Figure 3A, 3B). We found that the knockdown of TERT in BMSCs resulted in the reduction of osteogenic differentiation, as evidenced by the reduction of osteogenic differentiation markers OPN and Runx2 protein expression by western blotting (Figure 3C–3E) and the reduction of ALP staining as well as ALP activity (Figure 3F, 3G) compared to those in the NC group. We also found that the knockdown of TERT in BMSCs resulted in increased levels of apoptosis, as verified by the increased apoptosis ratios of BMSCs whose nuclei were labeled red, indicating increased apoptosis by Hoechst 33342/PI staining (Figure 3H, 3I), and the increased BAX protein expression and decreased Bcl-2 protein expression by western blotting compared to those in the NC group (Figure 3J–3L).

### LiCl reverses H_2_O_2_-induced inactivation of the Wnt pathway and inhibition of osteogenic differentiation in BMSCs

**Figure 4:**
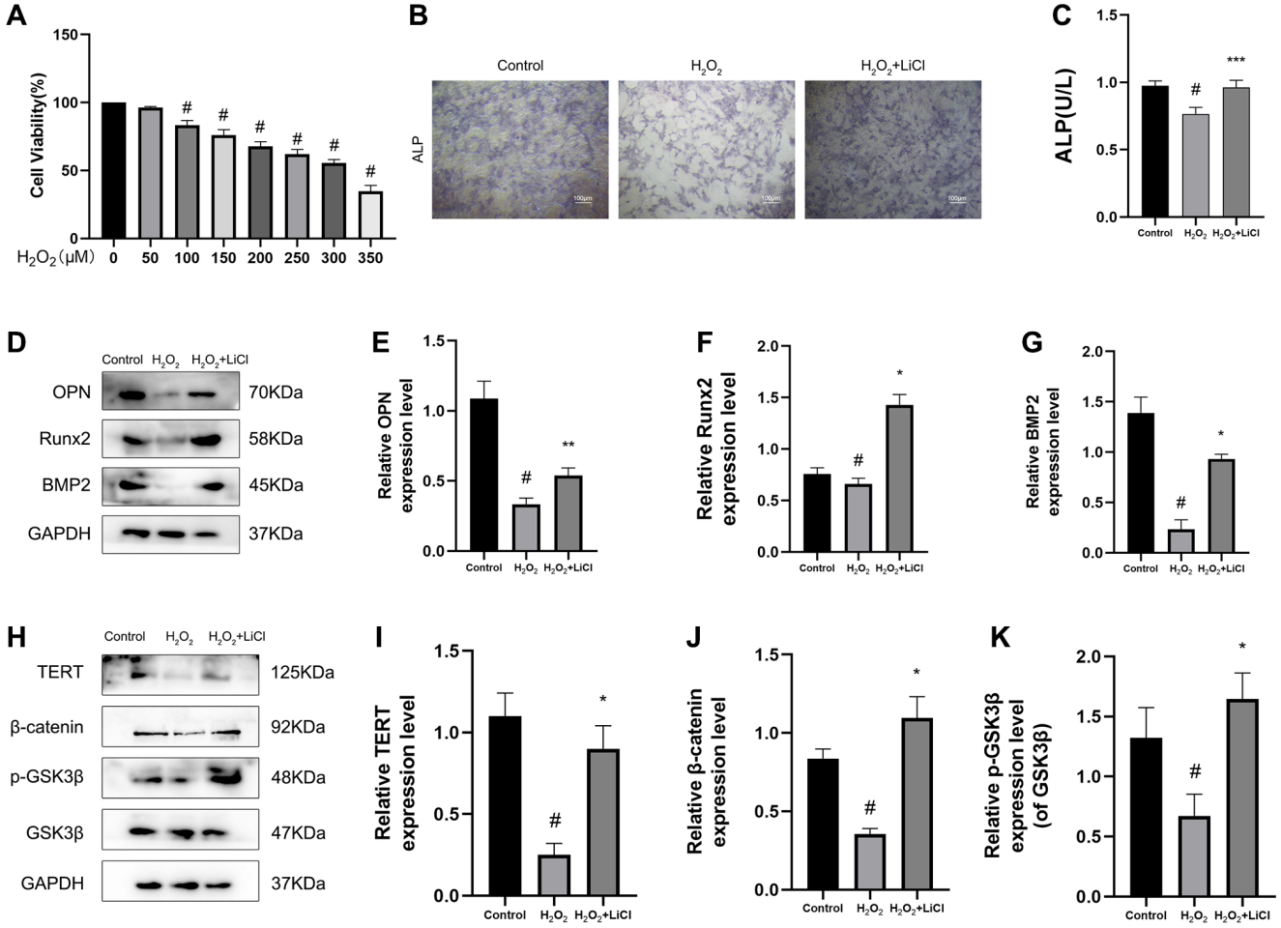
**LiCl reverses H_2_O_2_-induced inactivation of the Wnt pathway and inhibition of osteogenic differentiation in BMSCs.** (**A**) CCK-8 analysis was conducted to evaluate cell viability after being treated with different concentrations of H_2_O_2_ for 6 h. (**B**) ALP staining in three groups of BMSCs. (**C**) ALP activity of BMSCs detected by ALP activity assay kit. (**D**–**K**) Protein expression levels of TERT, β-catenin, p-GSK3β/GSK3β, OPN, Runx2, BMP2 in BMSCs treated with H_2_O_2_ or LiCl by Western blotting. All results are performed as mean ± SD. ^#^*P* < 0.05 vs. Control group. ^*^P < 0.05, ^**^P < 0.01, ^***^P < 0.005 vs. H_2_O_2_ group, *n* = 6.

H_2_O_2_ application to BMSCs mimics oxidative stress during the development of osteoporosis. To determine the optimal concentration of H_2_O_2_ acting on BMSCs, a CCK8 kit was used to detect the effect of different concentrations of H_2_O_2_ on the cell viability of BMSCs, and the results showed that 300 μM H_2_O_2_ could reduce the cell viability of BMSCs to 53.8%. From this, in the following experiments, 300 μM H_2_O_2_ was used as the damage concentration, and 4 hours of induction was used to cause cell apoptosis. (Figure 4A). In terms of osteogenic differentiation, ALP staining showed that the alkaline phosphatase staining of BMSCs was weaker and ALP activity was lower in the H_2_O_2_ group than in the control group, and the application of LiCl reversed these changes (Figure 4B, 4C). Consistent with the above results, the protein expression levels of the osteogenic-related markers OPN, Runx2, and BMP2 in BMSCs under the application of H_2_O_2_ were significantly reduced, as measured by western blotting, while the protein levels of the osteogenic-related markers showed the opposite trend after applying LiCl (Figure 4D–4G). We also found that TERT, β-catenin protein expression levels, and the p-GSK3β/GSK3β ratio, as measured by western blotting, were decreased in H_2_O_2_-induced BMSCs and that the application of LiCl reversed these changes (Figure 4H–4K).

### LiCl reverses H_2_O_2_-induced elevation of apoptosis in BMSCs

**Figure 5:**
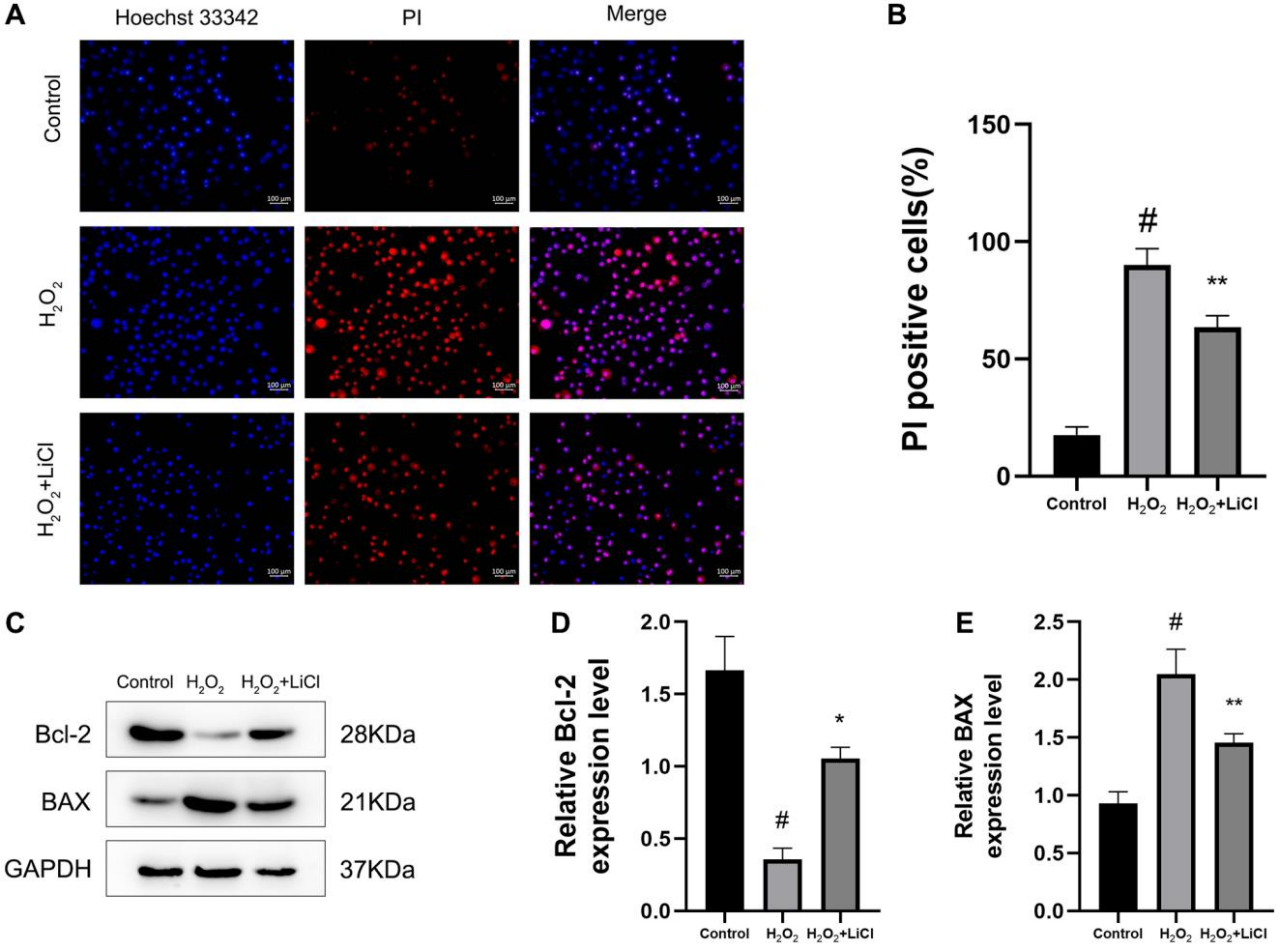
**LiCl reverses H_2_O_2_-induced elevation of apoptosis in BMSCs.** (**A**, **B**) The Hoechst 33342/PI and quantification results of apoptosis of BMSCs treated with H_2_O_2_ or LiCl. (**C**–**E**) Western blotting and quantification of Bcl-2 and BAX in BMSCs treated with H_2_O_2_ or LiCl. All results were performed as mean ± SD. ^#^*P* < 0.05 vs. control group, ^*^P < 0.05, ^**^P < 0.01 vs. H_2_O_2_ group, *n* = 6. Scale bar: 100 μm.

Hoechst 33342/PI staining results showed that the application of H_2_O_2_ significantly increased the apoptotic level of cells compared to that in the control BMSCs, while their apoptotic level was reduced after the application of LiCl (Figure 5A, 5B). Then, we examined the apoptotic status of BMSCs by western blotting, and the results showed that the expression of the anti-apoptotic protein Bcl-2 was decreased and the expression of the pro-apoptotic protein BAX was increased in the H_2_O_2_-induced group, while the application of LiCl changed the above result toward the opposite trend (Figure 5C–5E).

### LiCl reverses the H_2_O_2_-induced reduction in the nuclear translocation of β-catenin and the interaction between β-catenin and TERT in BMSCs

**Figure 6:**
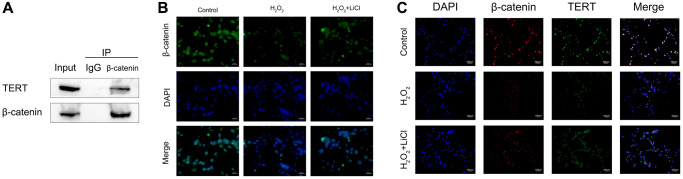
**LiCl reverses the H_2_O_2_-induced reduction in the nuclear translocation of β-catenin and the interaction between β-catenin and TERT in BMSCs.** (**A**) Co-IP showing β-catenin binding to TERT in BMSCs. (**B**) Immunofluorescence staining reveals nuclear translocation of β-catenin in BMSCs treated with H_2_O_2_ or LiCl. (**C**) Immunofluorescence assays were performed to identify colocalization between β-catenin and TERT in BMSCs treated with H_2_O_2_ or LiCl. All results were performed as mean ± SD. ^#^*P* < 0.05 vs. control group, ^*^P < 0.05 vs. H_2_O_2_ group, *n* = 6. Scale bar: 100 μm.

It is known that β-catenin nuclear translocation promotes the expression of target genes, such as Runx2, by binding to transcription factors such as TCF-4 or LEF-1. In the present study, we first verified the interaction between β-catenin and TERT in normal BMSCs by using an immunoprecipitation assay (Co-IP) (Figure 6A). Then, the immunofluorescence assay showed that β-catenin fluorescence intensity was weaker in the nuclei of BMSCs in the H_2_O_2_-induced group than in the control group, whereas it was darker in the nuclei after the application of LiCl, indicating that the application of LiCl could reverse the reduction in β-catenin nuclear translocation in BMSCs caused by H_2_O_2_ (Figure 6B). To investigate the effect of LiCl on the interaction between β-catenin and TERT, we then used immunofluorescence colocalization experiments, and the results showed that the fluorescence overlap fraction of β-catenin and TERT was reduced in BMSCs in the H_2_O_2_-induced group compared to that in the control group, while the application of LiCl significantly increased this fraction (Figure 6C). The above results suggest that LiCl reverses the reduction in the interaction between β-catenin and TERT caused by the application of H_2_O_2_ in BMSCs.

### Confirmation of the GSK3β/β-catenin/TERT pathway in BMSCs

**Figure 7:**
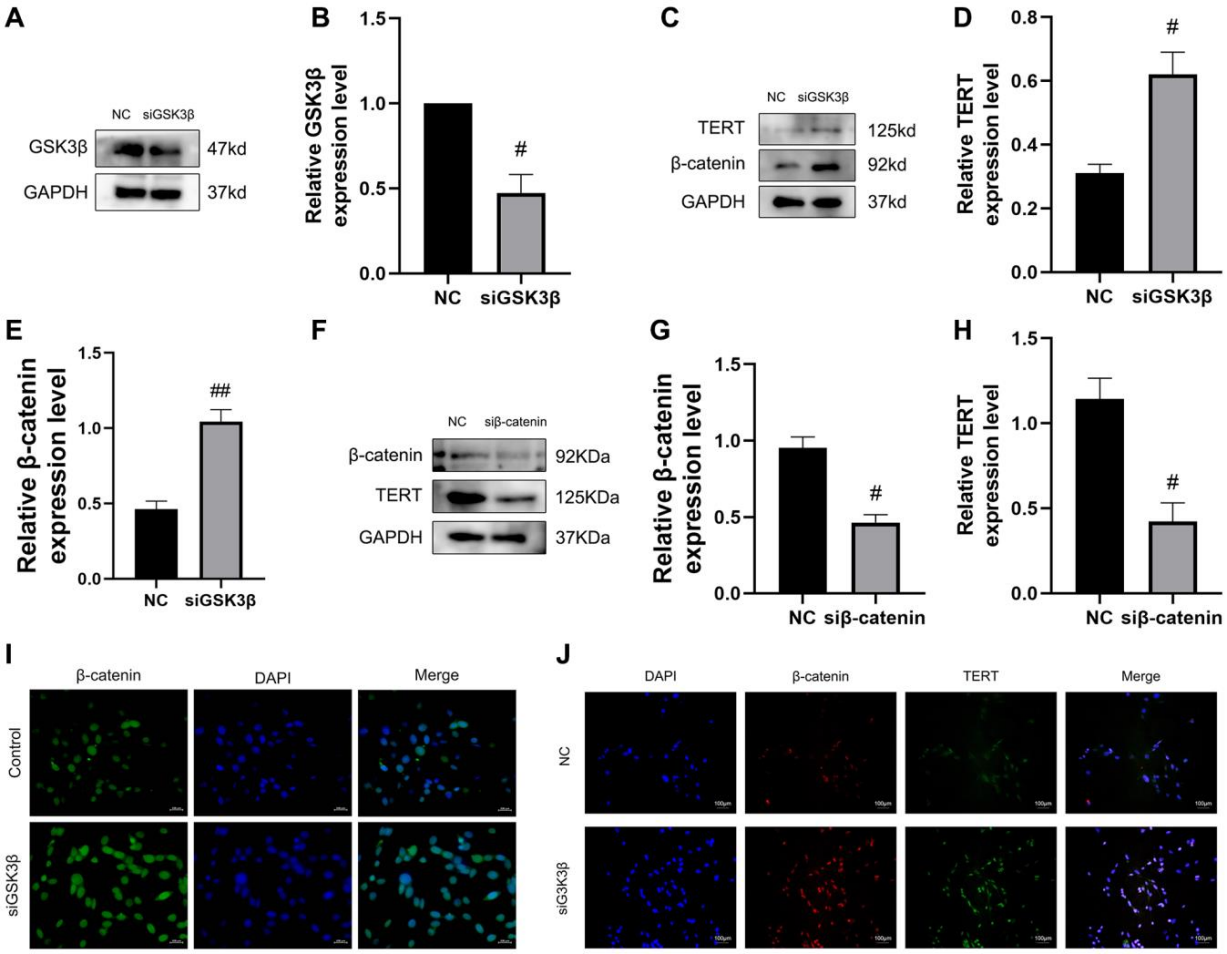
**Confirmation of the GSK3β/β-catenin/TERT pathway in BMSCs.** (**A**, **B**) The siRNA-mediated depletion of GSK3β was assessed by western blotting. (**C**–**E**) The specific proteins related to the pathway protein in this experiment expressed from the NC group and siGSK3β group of BMSCs were detected by Western blotting, including β-catenin and TERT. (**F**–**H**) The siRNA-mediated depletion of β-catenin and protein levels of TERT was assessed by western blotting from the NC group and siβ-catenin of BMSCs. (**I**) Immunofluorescence staining reveals nuclear translocation of β-catenin in BMSCs after depletion of GSK3β. (**J**) Immunofluorescence assays were performed to identify colocalization between β-catenin and TERT in BMSCs after depletion of GSK3β. All results were performed as mean ± SD. Scale bar: 100 μm. ^#^*P* < 0.05, ^##^*P* < 0.01 vs. NC group, *n* = 6.

Next, we confirmed the GSK3β/β-catenin/TERT pathway and verified the regulation of TERT expression by the Wnt pathway. First, we used small interfering RNA to knock down GSK3β in BMSCs, and western blotting verified the successful knockdown of GSK3β (Figure 7A, 7B). Western blotting results revealed that the protein expression of β-catenin and TERT was significantly increased in the siGSK3β group compared to that in the NC group (Figure 7C–7E). To further verify the upstream and downstream relationship between β-catenin and TERT, we used small interfering RNA to knock down β-catenin in BMSCs, and the results showed that knockdown of β-catenin in BMSCs led to a reduction in TERT protein expression by western blotting (Figure 7F–7H). The immunofluorescence assay showed that β-catenin fluorescence intensity was significantly increased in the nuclei of BMSCs in the siGSK3β group compared to that in the NC group, and the results of immunofluorescence colocalization experiments showed that the interaction between β-catenin and TERT was increased in the siGSK3β group compared to that in the NC group (Figure 7I, 7J). The above results validated the interrelationship of the GSK3β/β-catenin/TERT pathway.

### The Wnt/β-catenin pathway promotes osteogenic differentiation and inhibits the apoptosis of BMSCs through upregulating TERT expression

**Figure 8:**
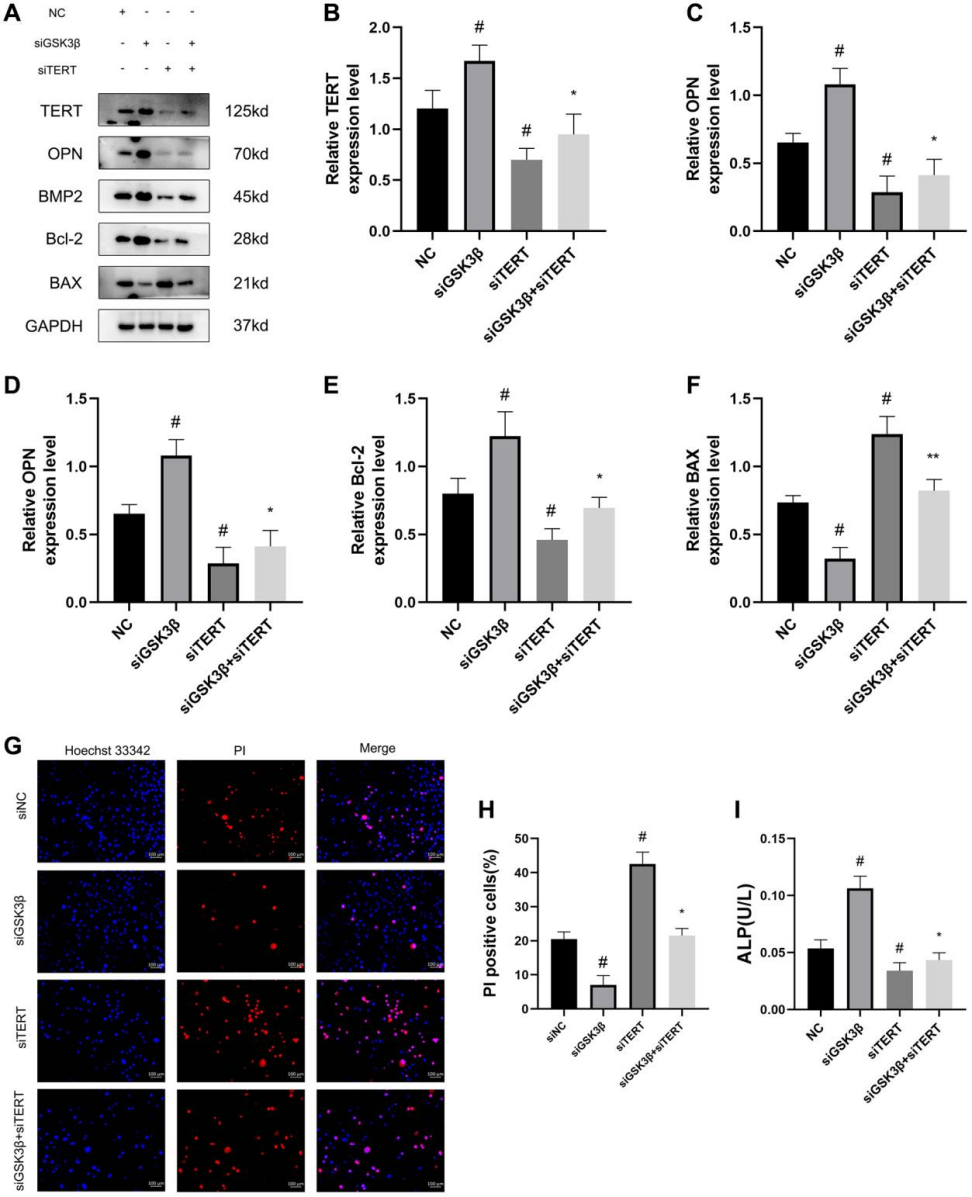
**The Wnt/β-catenin pathway promotes osteogenic differentiation and inhibits the apoptosis of BMSCs through upregulating TERT expression.** (**A**–**F**) Western blotting and quantification of TERT, OPN, BMP2, Bcl-2, and BAX in NC, siGSK3β, siTERT, and siGSK3β + siTERT groups of BMSCs. (**G**, **H**) The Hoechst 33342/PI and quantification results in four groups of BMSCs. (**I**) ALP activity assay kit to detect ALP activity in four groups of BMSCs. Scale bar: 100 μm. All results were performed as mean ± SD. ^#^*P* < 0.05 vs. NC group, ^*^P < 0.05 vs. siGSK3β group. *n* = 6.

To further verify the role of TERT in the osteogenic differentiation and apoptosis of BMSCs under the regulation of the Wnt pathway, we used small interfering RNA to simultaneously knock down the GSK3β and TERT genes in BMSCs. The cells were divided into four groups: NC, siGSK3β, siTERT, and siGSK3β+siTERT. Western blotting results showed that the TERT expression level in BMSCs was significantly elevated in the siGSK3β group and decreased in the siTERT group compared to that in the NC group, while it was slightly elevated in the siGSK3β+siTERT group compared to that in the siTERT group but lower than that in the siGSK3β group. Moreover, the expression trends of the osteogenic markers OPN and BMP2 and the antiapoptotic protein Bcl-2 were the same as those of TERT, and the expression trend of the apoptotic protein BAX was opposite to that of TERT (Figure 8A–8F). The results of Hoechst 33342/PI staining were also consistent with the above results. Compared to that in the NC group, the rate of apoptotic cells was reduced in the siGSK3β group and increased in the siTERT group, while the rate of apoptotic cells in the siGSK3β+siTERT group was slightly higher than that in the siGSK3β group but lower than that in the siTERT group (Figure 8G, 8H). The ALP activity, which was positively correlated with the osteogenic differentiation of BMSCs, was also consistent with the above results of the ALP activity assay (Figure 8I). The above results indicated that siTERT partially counteracted the osteogenic-promoting effect of siGSK3β in BMSCs, suggesting that the Wnt pathway promoted osteogenic differentiation and inhibited apoptosis in BMSCs and that the regulatory effects acted partly through upregulation of TERT expression.

## DISCUSSION

Osteoporosis is the most common skeletal disease in the world, with up to millions of fragility fractures caused by osteoporosis each year worldwide, and the root cause of the disease is the imbalance in bone turnover in the body [28]. Tissue engineering research has also proved the development of bone defect repair materials for osteoporosis-related fractures [29, 30]. In addition, relevant studies have shown that engineered exosomes containing bone-targeting drugs such as siRNAs and Wnt pathway activators can prevent bone loss and the progression of osteoporosis [31, 32]. Therapeutic agents for clinically diagnosed osteoporosis include bisphosphonates, selective estrogen receptor modulators, and hormone replacement therapy, but the side effects and long-term efficacy of the drugs have yet to be proven, limiting their use in clinical practice [33, 34]. Therefore, further research is needed to uncover the medical targets and specific regulatory mechanisms underlying osteoporosis, providing new directions and new theoretical support for the development of osteoporosis-delaying and therapeutic drugs.

TERT is a key catalytic subunit of telomerase, and its expression level is comparable to telomerase function. In recent years, research on TERT has mainly focused on biological processes such as tumorigenesis and immune inflammation [35–37]. In the field of stem cell osteogenic differentiation research, it has been found that the cells with the highest TERT activity measured in bone tissue are osteoblasts, and it has also been confirmed that the longer the telomeres in osteoblasts are, the faster the cell proliferation rate [38]. The Wnt pathway plays an irreplaceable role in determining the differentiation of various stem cells to osteoblast lineages by accelerating osteogenesis and reducing bone resorption, and Wnt inhibitors such as sclerostin inactivate the Wnt pathway and can reduce osteoblast differentiation [39, 40]. However, the specific regulatory mechanisms of TERT in OP and the interaction between the Wnt/β-catenin signaling pathway and TERT are not well understood. Therefore, this experiment focused on the role of TERT in regulating osteogenic differentiation, apoptosis, and the development of osteoporosis in BMSCs and whether the Wnt pathway has a regulatory effect on TERT.

*In vitro*, we knocked down TERT with small interfering RNA and found that the osteogenic differentiation ability of BMSCs was reduced and the level of apoptosis was increased, verifying that TERT played a positive regulatory role in the osteogenic differentiation and apoptosis of BMSCs. We further found that the p-GSK3β/GSK3β ratio and β-catenin and TERT protein expression were decreased in H_2_O_2_-induced BMSCs. The application of LiCl, an inhibitor of GSK3β, activated the Wnt signaling pathway and reversed the above changes, suggesting that TERT might play an important role in regulating stem cell osteogenic differentiation and apoptosis and that the Wnt signaling pathway plays a regulatory role in TERT expression. We finally used small interfering RNA to knock down GSK3β, β-catenin, and TERT in cells separately or simultaneously, verified the existence of the GSK3β/β-catenin/TERT pathway, and found that the Wnt/β-catenin pathway modulated stem cell osteogenic differentiation and apoptosis partially by regulating TERT expression. Our *in vivo* experiments also demonstrated the presence of the GSK3β/β-catenin/TERT pathway and its regulatory effects on BMSC osteogenic differentiation and apoptosis.

Our findings, that TERT promotes osteogenic differentiation of BMSCs, are consistent with those of Li et al., who found that MSCs transfected with TERT had enhanced proliferation and differentiation, especially osteogenic differentiation [41]. A previous study reported that found that the systemic transplantation of human mammary stem cell-derived exosomes in OVX mice improved bone loss in devitalized mice by upregulating TERT mRNA levels and elevating telomerase activity in BMSCs, thereby enhancing osteoblast differentiation [42]. Chen et al. [43] found that TERT could bind to β-catenin and BRG1, and the three genes interacted to assemble into a transcriptional complex binding precisely at the FAS ligand FAS ligand (FASL) promoter, where FASL transcription increased in abundance and the immune function of BMSCs was enhanced while knocking down TERT in BMSCs decreased their osteogenic differentiation, which was also consistent with our results.

Among the available experimental results, it has been demonstrated that TERT plays a pivotal role in regulating the differentiation of BMSCs toward the fate of the osteogenic lineage and attenuating the degree of apoptosis and that the Wnt pathway regulates its expression. However, our experiments are deficient for various reasons: (i) Epigenetic modifications have been found to play a key regulatory role in stem cell differentiation, and β-catenin recruits histone methyltransferases to the promoter region of TERT by removing specific methylation markers and thus regulating TERT expression [44, 45]. However, the regulation of TERT by β-catenin in the present study did not involve methylation as an in-depth mechanism. (ii) Liu et al. [46] found that human TERT interacts with β-catenin by inducing epithelial-mesenchymal transition (EMT) and a cancer cell phenotype, and whether TERT has a regulatory effect on β-catenin expression in BMSCs was not explored in the present study. (iii) The results in this study only showed the effects of siTERT on BMSC osteogenesis and did not assess the effects of constructing the TERT overexpression plasmid on the osteogenic differentiation and apoptosis of BMSCs.

In conclusion, our experiments verified that TERT in BMSCs promoted their osteogenic differentiation and inhibited apoptosis, and that the Wnt pathway has a regulatory role in TERT expression. TERT might serve as a new therapeutic target in osteoporosis.

## MATERIALS AND METHODS

### Cell culture and treatment

The mouse bone marrow mesenchymal stem cells (BMSCs) line was obtained from Chuanqiu Biotechnology (Shanghai, China, Item No: M015). BMSCs were grown in DMEM/F12 essential medium including 1% penicillin and streptomycin (KeyGEN BioTECH, Nanjing, China) and supplemented with 10% fetal bovine serum (FBS) in 5% CO2 at 37°C according to the approved culture protocols for this cell line.

### Animal models and grouping

Thirty female C57BL/6J mice (8 weeks old, mean weight 18.5 g) were purchased from Chang Sheng Biological Company (Benxi, Liaoning). They were randomly divided into three groups: sham-operated group (control, *n* = 10), bilateral ovariectomy group (OVX, *n* = 10), and bilateral ovariectomy plus GSK3β inhibitor lithium chloride gavage group (OVX+LiCl, *n* = 10). The mice were anesthetized with 1% pentobarbital sodium intraperitoneally after two weeks of acclimatization, and the ovariectomy was started under aseptic conditions. Mice in the OVX+LiCl group were gavaged with LiCl (200 mg/kg/d, dissolved using ddH_2_O) [47, 48]. The mice in the control group and OVX group were gavaged with the same volume of ddH_2_O daily. All mammalian studies were approved by the Dalian Medical University Animal Care Committee (Dalian, China; Certificate of Conformity: No. AEE21084). After 8 weeks of OVX, all mice were euthanized with 1% pentobarbital sodium, and the serum and femoral tissues were taken for subsequent experiments.

### Human bone tissue

Bone tissue samples were placed in a liquid nitrogen tank immediately after acquisition. This method of preservation maintains the activity of BMSCs in the tissue and maintains the integrity of the bone tissue.

### Micro-computed tomography (micro-CT) analysis

The femoral tissues of three groups of mice were taken and scanned using a Micro-CT instrument (ZhongKeKaisheng, Guangzhou, China) to reconstruct 3D images of the mice femur. Images of the femoral trabecular area, femoral cortical area, and other levels were obtained based on 3D images. Trabeculae-related parameters were quantified, including bone volume fraction (BV/TV), trabecular number (Tb.N), femoral bone mineral density (BMD), trabecular thickness (Tb.Th), bone volume ratio of bone surface area (BS/BV), trabecular pattern factor (Tb.Pf), and trabecular separation (Tb.Sp).

### H&E staining of mice femur

Mouse femurs were immersed in 4% paraformaldehyde fixative to deform protein coagulation and then placed in 10 % EDTA solution, decalcified at about 4 weeks, and the femurs were embedded in melted paraffin wax. Longitudinal sections 5 μm thick were cut, stained with hematoxylin-eosin, and scanned under an inverted microscope (Leica, Germany) after sectioning to derive histomorphological images of the femur.

### Mouse BMSCs osteogenic differentiation induction

BMSCs were uniformly seeded in the 6-well plates, and the osteogenesis was induced by adding an osteogenic induction solution after each group was molded. The osteogenic induction solution contained dexamethasone at a concentration of 100 nM, vitamin C at a concentration of 50 μM, and sodium β-glycerophosphate at a concentration of 10 mM. The osteogenic induction solution was changed every two days for 14 days for alkaline phosphatase staining.

### Western blotting

Western blotting was used to detect the expression of the relevant proteins in BMSCs or bone tissues. The protein of bone tissues was extracted as followings, firstly, full grinding of tissues by grinding apparatus, centrifugation after full soaking with protein lysis solution to obtain supernatant, and then the protein concentration was measured by BCA protein assay kit (Beyotime, China). The cellular protein is also obtained by protein lysis, and the steps of concentration measurement were the same as that of bone tissues. The obtained proteins were subjected to sampling, electrophoresis, gel cutting, membrane transfer, blocking, incubation of primary and secondary antibodies respectively, and development steps to finally obtain protein images and quantify the protein expression by Image J (National Institutes of Health, Bethesda, MA, USA) analysis software according to the manufacturer’s instructions. The primary antibodies used in this study were TERT (Bioss, China), β-catenin (Proteintech, China), GSK3β (Proteintech, China), p-GSK3β (Proteintech, China), RUNX2 (Abcam, American), BMP2 (Proteintech, China), OPN (Proteintech, China), Bcl-2 (Proteintech, China), BAX (Proteintech, China), and GAPDH (Proteintech, China). The horseradish peroxidase (HRP)-conjugated secondary antibodies (Proteintech, China) used were directed against primary antibodies.

### Alkaline phosphatase (ALP) staining and activity detection

To study the osteogenic ability of BMSCs, osteogenic induction was performed for 14 days after the end of cell modeling, and the assay of ALP activity in the cell lysate was performed according to the steps of the ALP activity kit (Nanjing Jiancheng Institute of Biological Engineering, China). In addition, ALP staining was also performed according to the alkaline phosphatase staining kit (Beyotime) instructions.

### Cell proliferation assay

Cell Counting Kit-8 (SEVEN, Beijing) was used to detect the effect of different concentrations of H_2_O_2_ on the cell viability of BMSCs. The steps were to spread the cells evenly in a 96-well plate, after which the cells were treated with different concentrations (0, 50, 100, 150, 200, 250, 300, 350, 400 μmol) of H_2_O_2_ for 4 hours, followed by changing the DMEM/F12 culture medium containing 10 μl CCK-8 to incubate the cells for 3 hours. Finally, the absorbance values at 450 nm per well were detected in a microplate reader (Thermo-354, Thermo Fisher Scientific), and cell viability was calculated according to the formula.

### Hoechst 33342/PI staining

BMSCs were evenly seeded in 24-well plates, and the culture solution was aspirated after the end of cell modeling, 0.8 to 1 ml of staining buffer (Beyotime), 5μL Hoechst staining solution and 5μL PI staining solution (Solarbio, Beijing) was added, incubated for 30 min at 4°C, and washed once with PBS solution and observed under an inverted microscope (Leica, Germany).

### Immunofluorescence staining of bone tissue and cells

The paraffin sections of the mouse femur were placed in an oven at 60°C for one day, removed and immersed in different concentrations of alcohol (95%, 75%, 50%, and 25%) and water for 5 minutes each, and then placed in 0.01 M sodium citrate solution and boiled in a microwave oven. After the above steps, the sections were rinsed with phosphate buffer, and then primary antibody (diluted 1:50-1:200) was added dropwise on the surface of the sections and incubated at 4°C in a refrigerator for 12–16 hours. On the second day, the sections were rinsed with phosphate buffer, and fluorescent secondary antibody (diluted 1:300) was added dropwise on the sections, which were closed at 37°C for 1 h. Then, the sections were incubated with DAPI solution for 10 min and protected from light, and finally photographed under a fluorescence microscope (Leica, Germany).

After modeling, BMSCs were washed using PBS, and then cells were fixed with 4% paraformaldehyde and washed again. Each well was perforated by adding 0.5% Triton-X 100 for 15 min, washed and then closed with 1% BSA for 30 min, and finally, the primary antibody (diluted 1:300) was added and hybridized at 37°C for 2 h. After PBS washing, the fluorescent secondary antibody (diluted 1:300) was added and incubated in a 37°C water bath for 1 h and were incubated with DAPI solution for 10 min. After that, the cells were washed again with PBS. Finally, it was photographed using a fluorescence microscope (Leica, Germany).

### Immunocoprecipitation (Co-IP)

The total protein was extracted from the cells and incubated with protein A/G Plus - agarose beads and conjugated with an anti-brief antibody. After incubation overnight at 4°C, the beads were centrifuged at 4°C, 500 × g for 5 min. The supernatant was discarded and the IP product was washed three times with PBS. The target proteins were finally detected by western blot analysis.

### BMSCs transfection

GSK3β, β-catenin, and TERT sequences were constructed by chemical synthesis, purchased from Gema Biologicals (Shanghai, China), and the sequences are shown in Table 1. BMSC was spread evenly on a 6-well plate, and the transfection was started when the cell density was about 50%. 0.5 OD of si or NC was added to 62. 5 μl of DEPC water, at which point si or NC was 20 mM, and the mate was diluted with DMEM/F12 complete medium to the same concentration as si or NC, and 1 ml of si/NC+mate was added to each well. After transfection for 4 to 6 h, the medium in each well was discarded, washed twice with complete medium, and then replaced with F12 complete medium, and waited for 2 to 3 days to extract the cell proteins for assay.

**Table 1:** siRNA sequence.

**Name**	**Sense (5′–3′)**	**Antisense (5′–3′)**
GSK3β Si-RNA	GGUUGCCAUCAAGAAAGUUTT	AACUUUCUUGAUGGCAACCTT
β-catenin Si-RNA	CCAGGUGGUAGUUAAUAAATT	UUUAUUAACUACCACCUGGTT
TERT Si-RNA	GGAAGAGUGUCUGGAGCAATTdTdT	UUGCUCCAGACACUCUUCCTT
NC	UUCUCCGAACGUGUCACGUTT	ACGUGACACGUUCGGAGAATT

### Statistical analysis

Statistical data analysis was completed using GraphPad Prism 7 and SPSS Statistics applications. All experimental data were calculated as mean (Mean) ± standard error (SD), and significant differences between any two groups were assessed by one-way analysis of variance (ANOVA), and *p*-values < 0.05 were considered as significant differences.

## Supplementary Materials

Supplementary Figure 1
