# Supplementary material for: The Wnt/β-catenin signaling pathway inhibits osteoporosis by regulating the expression of TERT: an in vivo and in vitro study
Source: Aging (Albany NY). 2023 Oct 19;15(20):11471–88. doi: 10.18632/aging.205136 (PMC10637795; doi:10.18632/aging.205136)
Supplement: Supplementary Figure 1 [file aging-15-205136-s001.pdf]

SUPPLEMENTARY FIGURE

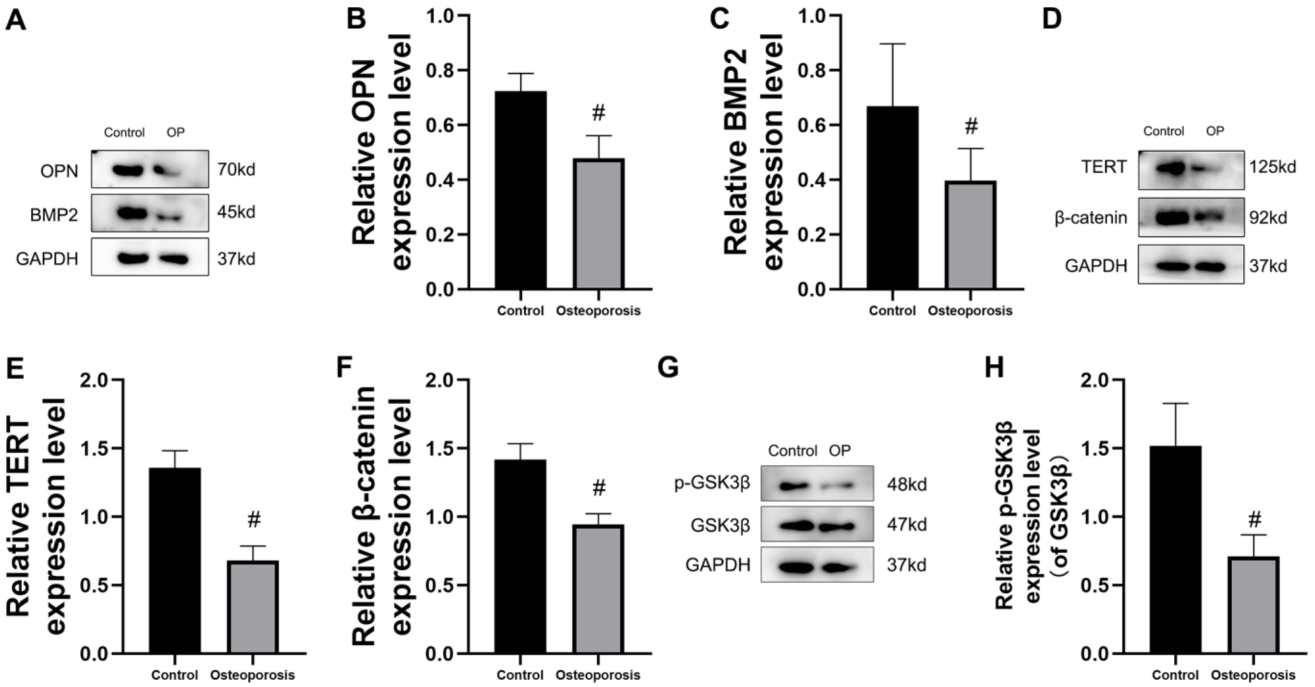

Supplementary Figure 1. Validation of target proteins in human bone tissues. (**A**–**C**) The specific proteins related to Osteogenesis differentiation expressed by human bone tissue were detected by Western blot, including OPN and BMP2. (**D**–**H**) The specific proteins related to the pathway protein in this experiment expressed by human bone tissue were detected by Western blot, including TERT, β-catenin, and p-GSK3β/GSK3β. All results were performed as mean ± SD. $^{\#}P < 0.05$ vs. control group. $n = 6$.
